# Changes in Cue Configuration Reduce the Impact of Interfering Information in a Predictive Learning Task

**DOI:** 10.3389/fpsyg.2016.02050

**Published:** 2017-01-06

**Authors:** Carmelo P. Cubillas, Miguel A. Vadillo, Helena Matute

**Affiliations:** ^1^Departamento de Psicología, Universidad a Distancia de MadridMadrid, Spain; ^2^Division of Health and Social Care Research, Department of Primary Care and Public Health Sciences, King's College LondonLondon, UK; ^3^Departamento de Psicología Básica, Universidad Autónoma de MadridMadrid, Spain; ^4^Facultad de Psicología y Educación, Universidad de DeustoBilbao, Spain

**Keywords:** configural learning, context, interference, renewal, information retrieval, predictive learning, associative learning

## Abstract

Decades of research in extinction and interference show that contexts can play a critical role at disambiguating the meaning of cues that have been paired with different outcomes at different times. For instance, if a cue *x* is followed by outcome 1 in the first phase of an experiment and by outcome 2 in a second phase, responses to cue *x* tend to be consistent with outcome 2 when tested in a context similar to that of the second phase of the experiment. However, if participants are taken back to the original context of the first phase (i.e., ABA renewal) or to a completely new context (i.e., ABC or AAB renewal), their responses to *x* tend to be more consistent with outcome 1. Although the role of physical and temporal contexts has been well studied, other factors that can also modulate the selective retrieval of information after interference have received less attention. The present series of experiments shows how changes in cue configuration can modulate responding in a similar manner. Across five experiments using a human predictive learning task, we found that adding, removing or replacing elements from a compound cue that had undergone an interference treatment gave rise to a recovery of responding akin to that observed after context changes in AAB renewal. These results are consistent with those of previous studies exploring the effect of changes of cue configuration on interference. Taken together, these studies suggest that a change in cue configuration can have the functional properties of a context change, a finding with important implications for formal models of configural learning and for classical accounts of interference and information retrieval.

## Introduction

Learning is an essential skill for survival. If a cue is consistently followed by an outcome, learning will allow an animal to predict this outcome and act accordingly. However, circumstances may change and the responses to a cue that were optimal in a specific situation can be damaging in a different setting. Fortunately, learning is a flexible mechanism that allows organisms to adapt their behavior to new situations. A typical procedure for studying how organisms adapt their response to new conditions is to present a signal followed consistently by an outcome (e.g., *x-o1*) and then, once animals have learned this association, present the same signal followed by a different outcome (e.g., *x-o2*). Not surprisingly, when presented with that cue, participants initially respond according to *o1* but then they adapt their responses to *o2*. Some of the most popular effects explored by learning and memory psychologists, like extinction (Pavlov, 1927; Miller et al., 2015), latent inhibition (Lubow, 1973; De la Casa and Lubow, 1995; Rodríguez et al., 2014; Miguez et al., 2015), counterconditioning (Bouton and Peck, 1992; Raes and De Raedt, 2012), or retrieval-induced forgetting (Anderson et al., 1994; Vadillo et al., 2013, 2016) belong to this family of interference phenomena.

Traditional associative learning models (e.g., Rescorla and Wagner, 1972) predict that, in these conflicting situations, the information acquired during the second phase should erase and replace the information learned in the first phase. This prediction, known as *catastrophic interference* (McCloskey and Cohen, 1989; Ratcliff, 1990; Lewandowsky, 1991), implies that the first information learned becomes irretrievable after second-phase training. However, a plethora of research shows that first-learned information is not usually erased from memory after an interference treatment: It remains encoded and can be retrieved through specific manipulations. For instance, Pavlov (1927) showed that, after an interference treatment, the mere passage of time elicited behavior consistent with the first information learned (i.e., spontaneous recovery). Recent research shows that changing the physical context immediately before testing can also cause the expression of behavior consistent with the first-learned information. This effect, known as *renewal*, can be observed when the two training phases and test are conducted in three different contexts (ABC renewal; Thomas et al., 2003; Pineño and Miller, 2004), when both training phases take place in the same context but the test is conducted in a new context (AAB renewal; Bouton and Ricker, 1994; Thomas et al., 2003; Rosas et al., 2006b), and, finally, when initial training and test are conducted in the same context but the second information is learned in a different context (ABA renewal; Bouton and Bolles, 1979; Rosas and Bouton, 1998; Paredes-Olay and Rosas, 1999).

In the present series of experiments we explored yet another factor that could also modulate the expression of information after an interference treatment: The configuration of the cues. Published data suggest that changes in the cues can affect the perception of contexts (e.g., Bouton and Swartzentruber, 1991; Lovibond et al., 2000). According to this hypothesis, a change in the configuration of the cue might induce the expression of the first information learned after an interference paradigm. Consistent with this prediction, a recent series of experiments using a fear conditioning preparation (Vervliet et al., 2004, 2005) has shown that this kind of manipulation can produce an effect similar to the ABA renewal. For example, during the first phase of the experiment, Vervliet et al. (2005) exposed their participants to repeated pairings of a geometric figure with a mild electric shock. In the second phase, for half of the participants the configuration of the cue changed (e.g., the angles of the shape became sharper), while for the other half the cue configurations remained as in the first phase. In either case, the cue was no longer followed by the shock in the second phase. At test, all participants were exposed to the cue configuration trained in the first phase. A significant increase in skin conductance was observed, but only in the group that experienced configuration changes across the experiment, suggesting that these participants expected the electric shock after the cue.

In these studies, changes in cue configuration were made by changing the orientation or position of the elements of the cue, resulting in different types of triangles or squares. However, other manipulations like adding, hiding, or changing elements of the cue, have been used as well. For example, in a within-subject design, Vervliet et al. (2007) paired two independent cues with an outcome during the first phase and then, during the second phase, they presented these two cues in compound several times without the outcome. At test, the single cues were presented again. The results show that the response acquired during first phase was retrieved. A similar approach has been used in studies that explore protection from extinction (e.g., Soltysik et al., 1983). In these studies the target cue is presented during interference with an inhibitory (Baum and Jacobs, 1989), an excitatory (Lovibond et al., 2000) or a neutral stimulus (Kamin, 1968), which reduces the impact of the interference phase at test, when the cue is presented again alone.

In all the previous examples, changes in cue configuration produced a retrieval of first-learned information in a situation akin to ABA renewal. That is, in all cases the configuration of the cue was the same during the first phase and at test, but different from the cue configuration presented during the second phase. If changes in cue configuration have the same functional properties as changes in the physical context, then it should be possible to observe other types of renewal (e.g., AAB or ABC) by means of changes in the configuration of the target cues. However, to the best of our knowledge, these effects remain unexplored. Some studies suggest that ABA renewal is stronger than ABC and AAB renewal (e.g., Nakajima et al., 2000; Tamai and Nakajima, 2000; Thomas et al., 2003), which could explain why there are no published data showing effects equivalent to ABC or AAB renewal with cue-configuration manipulations.

The purpose of the present series of experiments is to explore whether changes in cue configuration at test can produce the renewal of first-learned information in a human predictive learning task. In Experiments 1A–1C, the configuration of the target cue was changed at test by presenting alone a cue that had been trained in compound with another cue during both training phases. This manipulation is akin to the AAB renewal observed in experiments manipulating physical contexts (cf., Bouton and Ricker, 1994; Thomas et al., 2003; Rosas et al., 2006b). As explained above, we decided to explore this kind of renewal because other manipulations, such as ABA renewal, have already been found in experiments manipulating cue configuration (e.g., Lovibond et al., 2000; Vervliet et al., 2004, 2005). Also, as explained in the General Discussion, AAB renewal is more interesting than ABA renewal from a theoretical point of view, as it is more difficult to accommodate by traditional learning models (e.g., Rescorla and Wagner, 1972). In Experiments 2A and 2B we replicate the results of Experiments 1A and 1B and we test two additional changes in cue configuration: Adding and replacing elements from a compound cue.

## Ethics statement

The computer program informed participants that their participation was voluntary and anonymous. We did not ask participants for any data that could compromise their privacy, nor did we use cookies or software in order to obtain such data. The stimuli and materials were harmless and emotionally neutral, the goal of the study was transparent, and the task involved no deception. According to the U.S. Department of Health and Human Services (2009; Section 46.101b), as well as the American Psychological Association (2002; Section 8.05), no written informed consent is required under these circumstances. Even so, and in order to make sure that participants consented that the data they had generated during the experiment were used for research purposes, immediately after the study finished a screen asked them for permission to send us the data. Only the responses from those participants who clicked the button labeled “Send data” were submitted, stored, and used. Those participants not willing to submit their responses had the option of clicking a button labeled “Cancel,” which immediately deleted the data, so we did not receive it. The ethical review board of the University of Deusto examined and approved the procedure used in these experiments (Ref: ETK-44/12-13).

## Experiment 1A

In this experiment we tested the influence of a change in the configuration of the target cues on the expression of the first information learned in an interference paradigm. During the first stage, half of the participants were presented with repeated pairings of a compound cue with an outcome (i.e., *ax-o1*). Then, during the second stage, the same compound cue was followed by a new outcome (i.e., *ax-o2*). Finally, at test, only one element of the compound, *x*, was presented. Based on the theoretical framework discussed in the Introduction, we expected that the presentation at test of just one of the elements of the trained compound would give rise to the recovery of responses consistent with *o1*.

### Method

#### Participants and apparatus

One hundred and thirty three anonymous volunteers completed the task over the Internet through our laboratory's website (http://www.labpsico.deusto.es). The random allocation of participants to groups resulted in 70 participants in the Compound group and 63 in the Elemental group. The experimental task was embedded in a HTML website with JavaScript functions to control the presentation and timing of stimuli and the collection of data.

#### Procedure and design

The procedure was based on the Spy-Radio task (Pineño et al., 2000; Matute et al., 2007). In this task, participants are asked to imagine that they are soldiers that have to rescue war refugees. To save these refugees, participants are instructed to transport them to a safety zone using a truck. In some trials, the road can be mined. If the truck passes over a mine, all the refugees that were placed into the truck die. However, the truck is equipped with a Spy-Radio that can detect whether the road is mined or not. Different colors in the Spy Radio indicate the presence or absence of mines in the road. Participants are instructed to learn which colors predict that the road is free of mines and which colors predict that the road is mined.

**Figure 1:**
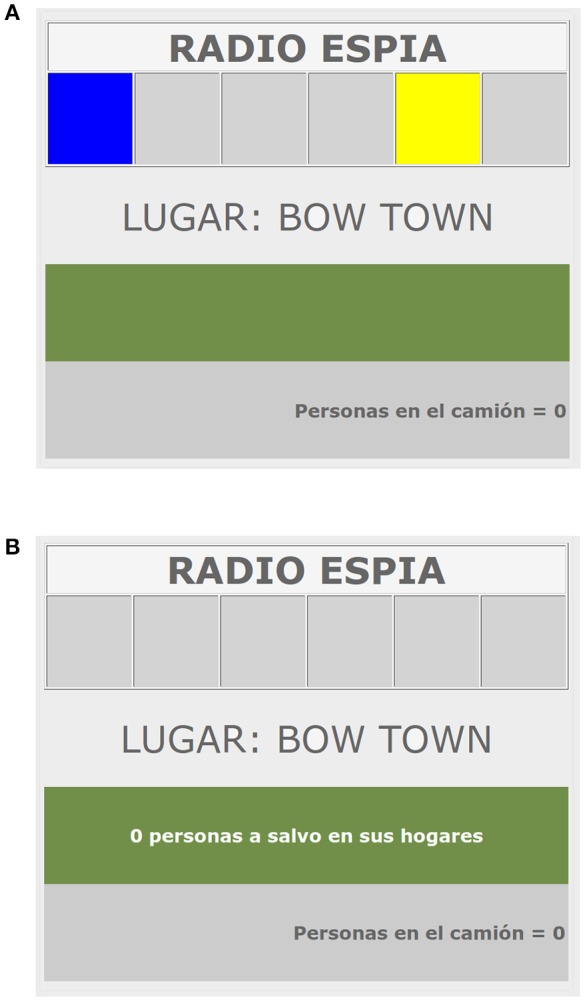
**Screenshot of a training trial in the Spy-Radio Task. (A)** Presentation of cues. The cues were presented in randomly chosen rectangles at the top of the screen. At the bottom of the screen participants could see the number of people they had placed into the truck. Participants could respond only during the 2 s, the interval in which the cues were present. After this period of time, the cues disappeared and the outcome screen was automatically presented. **(B)** Presentation of outcomes. Participants could see if the number of refugees they had placed into the truck survived (as in the example) or died.

At the beginning of each trial, the color cues are presented randomly in one (or two, in the case of compound cues) of the six panels of the Spy Radio and participants have to decide whether to press the space-bar to put refugees into the truck while the lights are on (see Figure 1). Each keystroke puts an additional refugee into the truck. If participants hold the space-bar pressed, the number of refugees in the truck increases much faster. After 2 s, the lights are turned off and participants are shown the outcome of the trial, namely, whether the road was mined or not. If the road was free of mines, the refugees placed into the truck in that trial are saved and participants win as many points as saved refugees. If the road was mined, all the refugees placed into the truck die and participants loose one point per refugee (for more information about the experimental task, including instructions, see Pineño et al., 2000).

The design of Experiment 1A is summarized in Table 1. In the Elemental group cue *x* was trained on its own in an interference paradigm [Elemental Interference (EI) condition]. Additionally, cue *y* was trained only in the first phase [Elemental Control (EC) condition]. Any difference in responding to *x* and *y* at test in this group can be used as a measure of interference. In the Compound group, cues *x* and *y* were always presented in compound with cues *a* and *b*, respectively. However, cue *x* was trained in an interference paradigm [Compound Interference [CI] condition], while cue *y* was presented only in the first phase [Compound Control [CC] condition)] At the end of the experiment, both *x* and *y* were tested on their own. The test consisted of a single presentation of cue *x* and a single presentation of cue *y*, counterbalancing the order across participants. Any evidence of reduced interference to *x*, in comparison with the elemental group, would suggest that the change in the configuration of the target cue, from *ax* to *x*, enhanced the retrievability of the associations trained in the first learning phase. As shown in Table 1, participants were also exposed to filler trials with other cues. These were included in the experiment to ensure that both groups were familiar with elemental and compound cues and to ensure that all the stages of the experiment included presentations of both *o1* and *o2*.

**Table 1:** **Design summary of Experiment 1A**.

		**Training**		
**Group**	**Condition**	**Phase 1**	**Phase 2**	**Test**
Compound	CI	**ax-o1 (16)**	**ax-o2 (16)**	**x?**
	CC	**by-o1 (16)**	–	**y?**
	Fillers	c-o2 (8)	c-o1 (16)	–
		de-o2 (8)	de-o2 (16)	
Elemental	EI	**x-o1 (16)**	**x-o2 (16)**	**x?**
	EC	**y-o1 (16)**	–	**y?**
	Fillers	c-o2 (8) de-o2 (8)	c-o1 (16) de-o2 (16)	**-**

*Conditions CI, EI, CC, and EC refer to Compound Interference, the Elemental Interference, the Compound Control, and the Elemental Control. Cues a-e and x-y denote different colors in the Spy-Radio, counterbalanced across participants. Outcomes o1 and o2 denote the absence or presence of mines in the road, respectively. The numbers in parentheses denote the number of times each type of trial was presented within a specific phase. Bold characters denote trials involving the target cues*.

Different colors of the Spy-Radio were used as cues. Blue, green, red and yellow were used as cues *x, y, a*, and *b*, counterbalanced across participants. For all participants, cues *c, d*, and *e* were brown, purple and black, respectively. In this and subsequent experiments, the absence of mines in the road (suggesting that it was safe to place refugees in the truck) always played the role of *o1*, and the presence of mines (suggesting that it was unsafe to place refugees in the truck) played the role of *o2*. Therefore, the number of refugees placed on the truck on any given trial can be used as a measure of the extent to which participants expected *o1*. During test trials, the cue was followed by a neutral outcome that did not allow participants to win or lose any points. The number of responses that participants gave in each trial was used as the dependent variable of the experiment.

#### Results and discussion

Following our usual data selection criterion with this task (e.g., Vadillo et al., 2006), we removed from the analyses data from 14 participants (7 from each group) who by the end of each training phase responded more to a cue paired with *o2* than to a cue paired with *o1*. Greenhouse-Geisser corrections were applied whenever Maunchy's test revealed a violation of the sphericity assumption.

**Figure 2:**
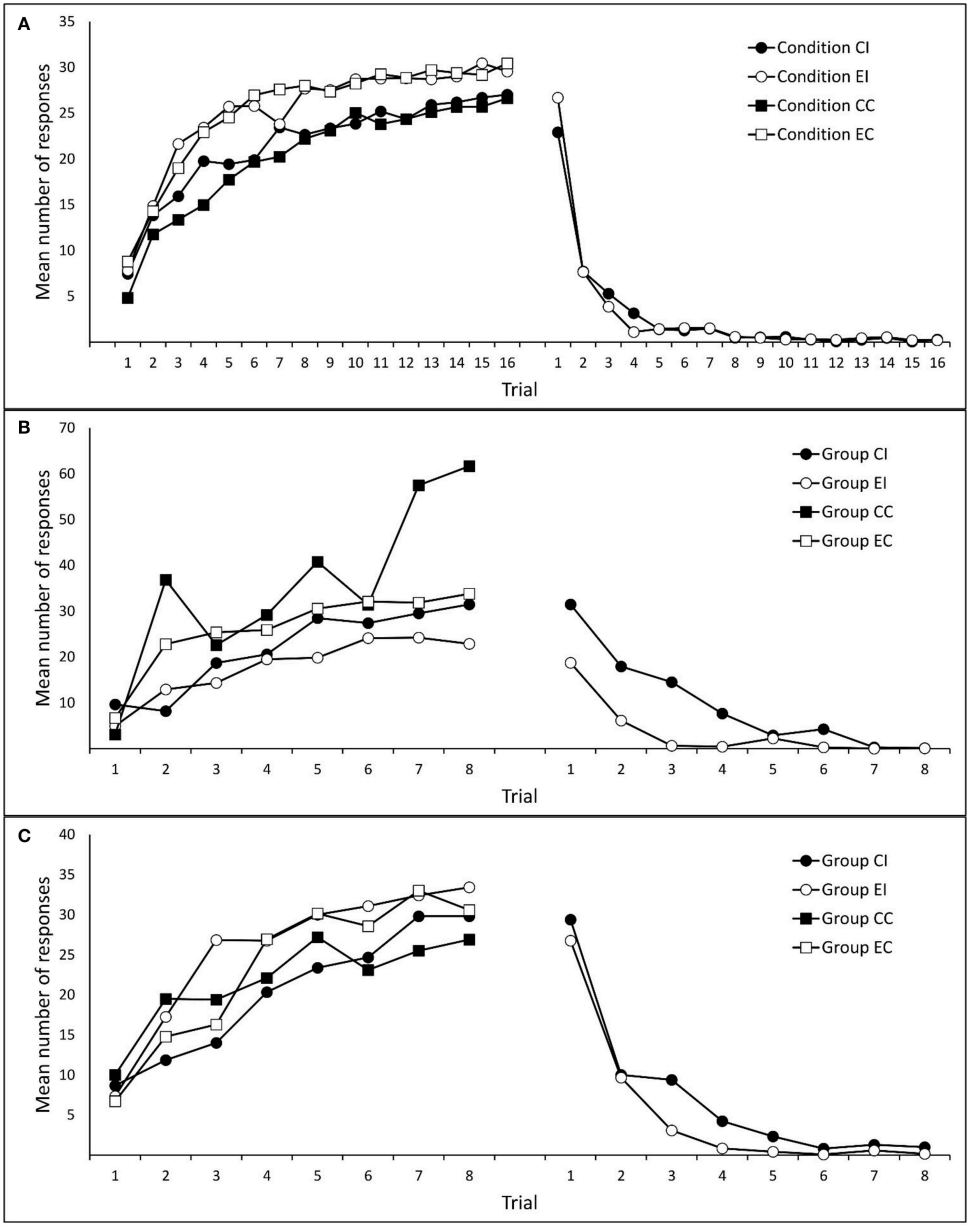
**Mean number of responses to the target cues (***ax***/***x***/***by***/***y***) during training**. Panels **(A–C)** refer to Experiments 1A, 1B, and 1C, respectively.

Figure 2A shows mean responses to cues *x* and *y* during Phases 1 and 2. A 2 (group: Compound vs. Elemental) × 2 (cue: *x* vs. *y*) × 16 (trial) mixed analysis of variance (ANOVA) on responses during Phase 1 yielded a significant effect of trial, *F*_(5.42, 634.66)_ = 105.18, *p* < 0.001, η_*p*_^2^ = 0.473, and an unexpected effect of group, *F*_(1, 117)_ = 8.23, *p* = 0.005, η_*p*_^2^ = 0.066. The rest of effects and interactions were non-significant, largest *F*_(15, 1755)_ = 1.52, *p*s > 0.088. These results confirm that, as expected, the number of responses to cues *x* and *y* increased progressively during Phase 1. For unknown reasons, participants in the Compound group showed a slightly lower level of performance. A 2 (group: Compound vs. Elemental) × 16 (trial) ANOVA on responses during Phase 2 yielded only a main effect of trial, *F*_(3.99, 467.06)_ = 162.00, *p* < 0.001, η_*p*_^2^ = 0.581. The main effect of group, *F*_(1, 117)_ = 0.019, *p* = 0.891, η_*p*_^2^ < 0.001, and the trial × group interaction, *F*_(15, 1755)_ = 1.48, *p* = 0.105, η_*p*_^2^ = 0.012, were non-significant.

**Figure 3:**
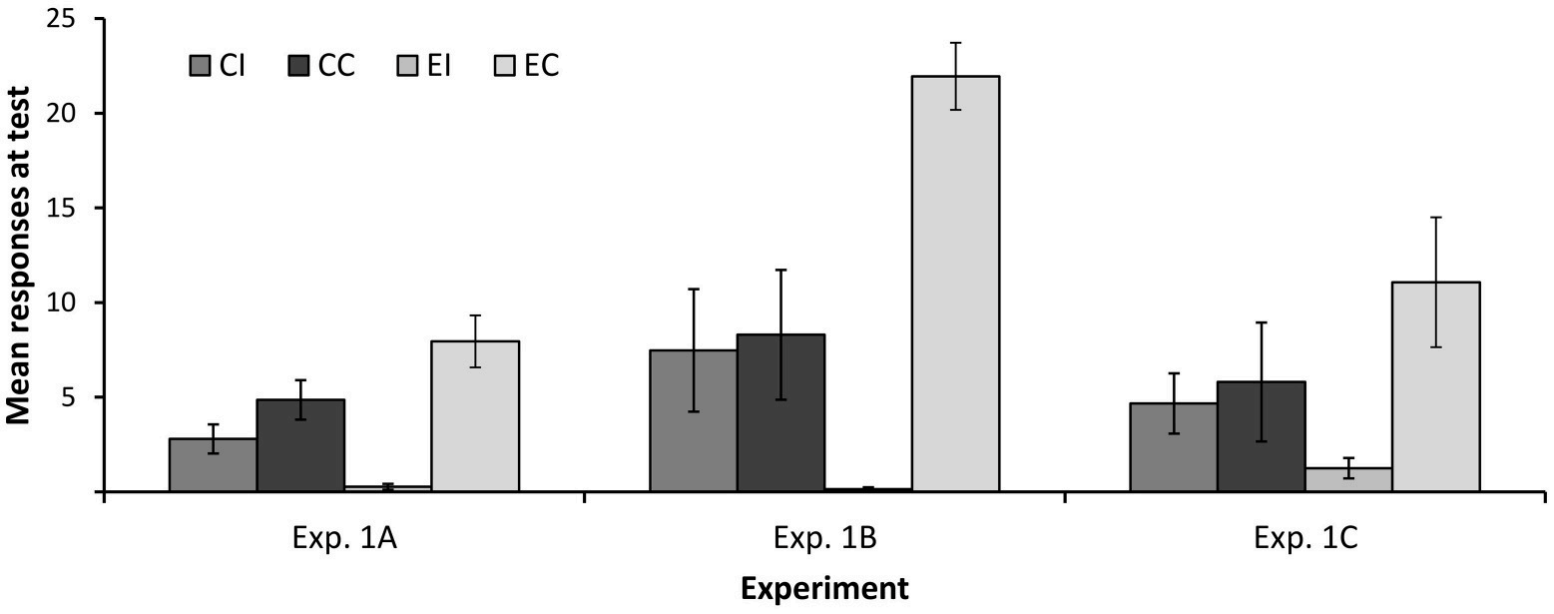
**Mean number of responses at test in Experiments 1A–1C**. Error bars denote standard errors of the mean.

Figure 3 depicts the mean number of responses to cues *x* and *y* at test. As can be seen, participants in both groups responded more to the control cue *y* than to cue *x*, suggesting some degree of interference in both groups. However, the difference in responding to *x* and *y* was larger in the elemental than in the compound group; that is, interference was stronger in the former than in the latter. Consistent with this, a 2 (type of training: Compound vs. Elemental) × 2 (condition: Interference vs. Control) ANOVA yielded a main effect of condition *F*_(1, 117)_ = 33.45, *p* < 0.001, η_*p*_^2^ = 0.222, and a significant interaction, *F*_(1, 117)_ = 11.11, *p* = 0.001, η_*p*_^2^ = 0.087. The main effect of type of training was far from statistical significance, *F* < 1. Overall, these results are consistent with the hypothesis that the change in the configuration of the target cues (from *ax* to *x*) at test facilitated the expression of the information originally learned during Phase 1, reducing the amount of interference.

## Experiment 1B

Experiment 1B was a conceptual replication of Experiment 1A. The only difference is that, unlike Experiment 1A, both experimental manipulations (interference vs. control and compound vs. elemental training) were conducted between participants. We expected that this minor modification would minimize the impact of carry-over effects among conditions, particularly during the test stage (e.g., reduced responding to the second test trial). Disentangling both manipulations also allowed us to reduce the length of the experiment and, consequently, the influence of fatigue or distractions on participants' performance. The design of Experiment 1B allowed us to ameliorate a potential shortcoming of Experiment 1A. As can be seen in Table 1, in the previous experiment filler cues were more likely to be paired with *o2* than with *o1*. This asymmetry might have encouraged participants to expect *o2* more strongly than *o1*, especially when faced with an unfamiliar configuration of cues, thereby hindering recovery of responding to the target cues. This feature of Experiment 1A works against the results we observed, which, if anything, makes them even more noteworthy. However, in Experiment 1B we used an alternative design that removed the influence of this potential confound.

### Method

#### Participants and apparatus

Sventy-nine psychology students from the University of Deusto volunteered to take part in the experiment in exchange for course credit. Random allocation of participants to each group resulted in 19 participants in group CI, 20 in group EI, 19 in group CC and 21 in group EC. Each participant completed the task in an individual cubicle.

#### Design and procedure

All details of the procedure where identical to Experiment 1A. As shown in Table 2, the critical cue, *x*, received a different treatment in each group. In groups CI and CC cue *x* was always trained in compound with *a*, while in groups EI and EC it was always trained on its own. Orthogonally, in two groups (CI and EI) cue *x* was paired with *o1* during Phase 1 and with *o2* during Phase 2, while in the other two groups (CC and EC) cue *x* was only paired with *o1* during Phase 1 and was not presented during Phase 2. In addition to the critical trials involving cue *x*, participants were also exposed to filler trials. At test, all groups were presented once with cue *x*.

**Table 2:** **Design summary of Experiment 1B**.

	**Training**		
**Group**	**Phase 1**	**Phase 2**	**Test**
CI	**ax-o1 (8)**	**ax-o2 (8)**	**x?**
	b-o2 (8)	d-o1 (8)	
EI	**x-o1 (8)**	**x-o2 (8)**	**x?**
	bc-o2 (8)	de-o1 (8)	
CC	**ax-o1 (8)**	f-o2 (8)	**x?**
	b-o2 (8)	d-o1 (8)	
EC	**x-o1 (8)**	f-o2 (8)	**x?**
	bc-o2 (8)	de-o1 (8)	

*Groups CI, EI, CC, and EC refer to the Compound Interference group, the Elemental Interference group, the Compound Control group, and the Elemental Control group. Cues a-f and x denote different colors in the Spy-Radio, counterbalanced across participants. Outcomes o1 and o2 denote the absence or presence of mines in the road, respectively. The numbers in parentheses denote the number of times each type of trial was presented within a specific phase. Bold characters denote trials involving the target cues*.

### Results and discussion

Following the same data selection criterion used in Experiment 1A, the data from 12 participants (2, 6, 2, and 2 from groups CI, EI, CC, and EC, respectively) were removed from subsequent analyses. Figure 2B depicts mean responding to cue *x* during Phases 1 and 2. A 2 (type of training: Compound vs. Elemental) × 2 (condition: Interference vs. Control) × 8 (trials) mixed ANOVA on responses during Phase 1, revealed only a significant main effect of trial, *F*_(1.18, 74.36)_ = 8.50, *p* = 0.003, η_*p*_^2^ = 0.119. The rest of effects and interactions were non-significant, although the main effect of condition approached marginal significance, *F*_(1, 63)_ = 2.51, *p* = 0.118, η_*p*_^2^ = 0.038, all other *F*s < 1.30. A 2 (type of training: Compound vs. Elemental) × 8 (trial) ANOVA on responses during Phase 2 (only in groups CI and EI) yielded a significant main effect of trial, *F*_(3.73, 108.08)_ = 30.89, *p* < 0.001, η_*p*_^2^ = 0.516, a significant main effect of type of training, *F*_(1, 29)_ = 18.84, *p* < 0. 001, η_*p*_^2^ = 0.394, and a significant interaction between both factors, *F*_(7, 203)_ = 3.76, *p* = 0.001, η_*p*_^2^ = 0.115.

Figure 3 shows the mean number of responses at test in each group. As in Experiment 1A, the difference between groups EI and EC reveals a strong interference. However, this difference is completely absent in groups CI and CC. These impressions were confirmed by a 2 (type of training: Compound vs. Elemental) × 2 (condition: Interference vs. Control) ANOVA on the number of responses at test, which yielded a main effect of condition, *F*_(1, 63)_ = 13.83, *p* < 0.001, η_*p*_^2^ = 0.180, and a significant condition × type of training interaction, *F*_(1, 63)_ = 11.89, *p* = 0.001, η_*p*_^2^ = 0.159. The main effect of type of training did not reach statistical significance, *F*_(1, 63)_ = 1.081, *p* = 0.303, η_*p*_^2^ = 0.017. These analyses show that the main results of Experiment 1A were replicated using a between-subjects design: A change in the configuration of the target cue between training and test completely abolished the interference that was observed in an otherwise similar condition.

## Experiment 1C

A potential shortcoming of Experiments 1A and 1B is that, in both cases, participants in the Elemental conditions could interpret the testing trial as just another trial of the training phase. However, for participants in the Compound conditions there was a clear break between the last training phase and test, because the cue presented at test (*x* or *y*, on their own) had never been presented during training. To avoid this potential confound, in Experiment 1C we inserted an additional training phase between Phase 2 and test. The target cue was never presented during Phase 3. We expected that this would equate all conditions (Compound and Elemental) in terms of perceiving a discontinuity between the test trial and the immediately preceding trials.

### Method

#### Participants and apparatus

Sixty psychology students from the University of Deusto volunteered to take part in the experiment in exchange for course credit. Random allocation of participants to each group resulted in 22 participants in group CI, 13 in group EI, 10 in group CC and 15 in group EC. The task was conducted in the same setting as Experiment 1B.

#### Design and procedure

Except for the inclusion of two additional colors (pink and orange) within the set of cues, the procedure was identical to Experiment 1B. As can be seen in Table 3, the design of the experiment was identical to Experiment 1B, except that all participants were exposed to 8 *g-o1* and 8 *h-o2* trials between Phase 2 and test.

**Table 3:** **Design summary of Experiment 1C**.

	**Training**			
**Group**	**Phase 1**	**Phase 2**	**Phase 3**	**Test**
CI	**ax-o1 (8)**	**ax-o2 (8)**	g-o1 (8)	**x?**
	b-o2 (8)	d-o1 (8)	h-o2 (8)	
EI	**x-o1 (8)**	**x-o2 (8)**	g-o1 (8)	**x?**
	bc-o2 (8)	de-o1 (8)	h-o2 (8)	
CC	**ax-o1 (8)**	f-o2 (8)	g-o1 (8)	**x?**
	b-o2 (8)	de-o1 (8)	h-o2 (8)	
EC	**x-o1 (8)**	f-o2 (8)	g-o1 (8)	**x?**
	bc-o2 (8)	de-o1 (8)	h-o2 (8)	

*Groups CI, EI, CC, and EC refer to the Compound Interference group, the Elemental Interference group, the Compound Control group, and the Elemental Control group. Cues a-h and x denote different colors in the Spy-Radio, counterbalanced across participants. Outcomes o1 and o2 denote the absence or presence of mines in the road, respectively. The numbers in parentheses denote the number of times each type of trial was presented within a specific phase. Bold characters denote trials involving the target cues*.

### Results and discussion

Following the same data selection criteria used in the previous experiments, data from three participants (one participant in each group except CC) were removed from subsequent analyses. Figure 2C shows participants' mean responses to cue *x* during Phases 1 and 2. A 2 (type of training: Compound vs. Elemental) × 2 (condition: Interference vs. Control) × 8 (trials) mixed ANOVA on responses to cue *x* during Phase 1 yielded only a main effect of trial, *F*_(4.74, 251.30)_ = 33.52, *p* < 0.001, η_*p*_^2^ = 0.387. The rest of effects and interactions were non-significant, largest *F*_(1, 53)_ = 2.22, *p*s > 0.142, η_*p*_^2^s > 0.040. Similarly, A 2 (type of training: Compound vs. Elemental) × 8 (trials) mixed ANOVA on responses to cue *x* during Phase 2 (only in the Interference condition), revealed only a main effect of trials, *F*_(2.96, 91.85)_ = 44.25, *p* < 0.001, η_*p*_^2^ = 0.588; all other *F*s < 1.

Mean responses to cue *x* in the test trial are shown in Figure 3. A 2 (type of training: Compound vs. Elemental) × 2 (condition: Interference vs. Control) between-subjects ANOVA yielded a main effect of condition, *F*_(1, 53)_ = 5.09, *p* = 0.028, η_*p*_^2^ = 0.088, but no main effect of type of training, *F* < 1. The crucial interaction approached statistical significance, *F*_(1, 53)_ = 3.20, *p* = 0.078, η_*p*_^2^ = 0.057. Although only marginally significant, this interaction is consistent with the results of Experiments 1A and 1B showing that retroactive interference is reduced when the configuration of the target cue changes between training at test.

Visual inspection of Figure 3 suggest that, despite the similarities in the designs of Experiments 1B and 1C, responding at test was noticeably lower in the latter. This is possibly due to the fact that the filler cues presented during Phase 3 are paired with the same outcomes used for the target cue *x*. It is well known that training a novel cue-outcome association hinders responding to other cues that have been paired with the same outcome in the past (i.e., interference between cues; see Escobar et al., 2002; Vadillo et al., 2008; Luque et al., 2009, 2011). This interference effect might have limited overall responding to *x* at test, which would explain why some of the key statistical contrasts failed to reach conventional levels of significance.

## Experiment 2A

The results of Experiments 1A–1C consistently supported the hypothesis that a change in the configuration of a cue (in this case, removing an element from a compound) can have the functional properties of a context change. After an interference treatment, changing the configuration of the cue increased responding consistent with the first information learned. In these three experiments, we manipulated the configuration of the target cue by presenting a compound cue during training and removing one of its elements at test. In the present experiment we tested whether a similar recovery of responding can be observed with alternative manipulations. Specifically, we assessed whether adding or replacing elements from the original cue configuration gives rise to the same pattern of results.

### Method

#### Participants and apparatus

One hundred and one psychology students from the University of Deusto volunteered to take part in the experiment. All participants completed the experimental task simultaneously in a large computer room. Random allocation of participants to the experimental conditions resulted in 26 participants in group *x*/*ax*, 24 in group *ax*/*x*, 24 in group *ax*/*abx*, and 27 in group *ax*/*bx*.

#### Procedure and design

The procedure was identical to Experiments 1A–1C. The design of the experiment is summarized in Table 4. As can be seen, three groups of participants were trained with the compound cue *ax* in Phases 1 and 2. These participants were then tested either on *x* alone, on a new compound with three cues, *abx*, or on a two-cue compound with *x* and a new cue, *bx*. Additionally, another group of participants was trained with cue *x* presented alone in every trial, and then tested with the compound *ax*. Unlike in Experiments1A–1C, in this and the following experiment we did not use a control group to assess the amount of interference. Instead we relied on a comparison between responses to cue *x* in the last training trial of Phase 2 and responses to cue *x* at test. An increase in responding with respect to the last trial of Phase 2 was interpreted as a retrieval of the information learned in Phase 1.

**Table 4:** **Design summary of Experiment 2A**.

	**Training**		
**Group**	**Phase 1**	**Phase 2**	**Test**
x/ax	**x-o1 (8)**	**x-o2 (8)**	**ax?**
	bc-o2 (8)	de-o1 (8)	
	f-o1 (4)	f-o1 (4)	
ax/x	**ax-o1 (8)**	**ax-o2 (8)**	**x?**
	c-o2 (8)	d-o1 (8)	
	fg-o1 (4)	fg-o1 (4)	
ax/abx	**ax-o1 (8)**	**ax-o2 (8)**	**abx?**
	c-o2 (8)	d-o1 (8)	
	fg-o1 (4)	fg-o1 (4)	
ax/bx	**ax-o1 (8)**	**ax-o2 (8)**	**bx?**
	c-o2 (8)	d-o1 (8)	
	fg-o1 (4)	fg-o1 (4)	

*Cues a-g and x denote different colors in the Spy-Radio, counterbalanced across participants. Outcomes o1 and o2 denote the absence or presence of mines in the road, respectively. The numbers in parentheses denote the number of times each type of trial was presented within a specific phase. Bold characters denote trials involving the target cues*.

### Results and discussion

**Figure 4:**
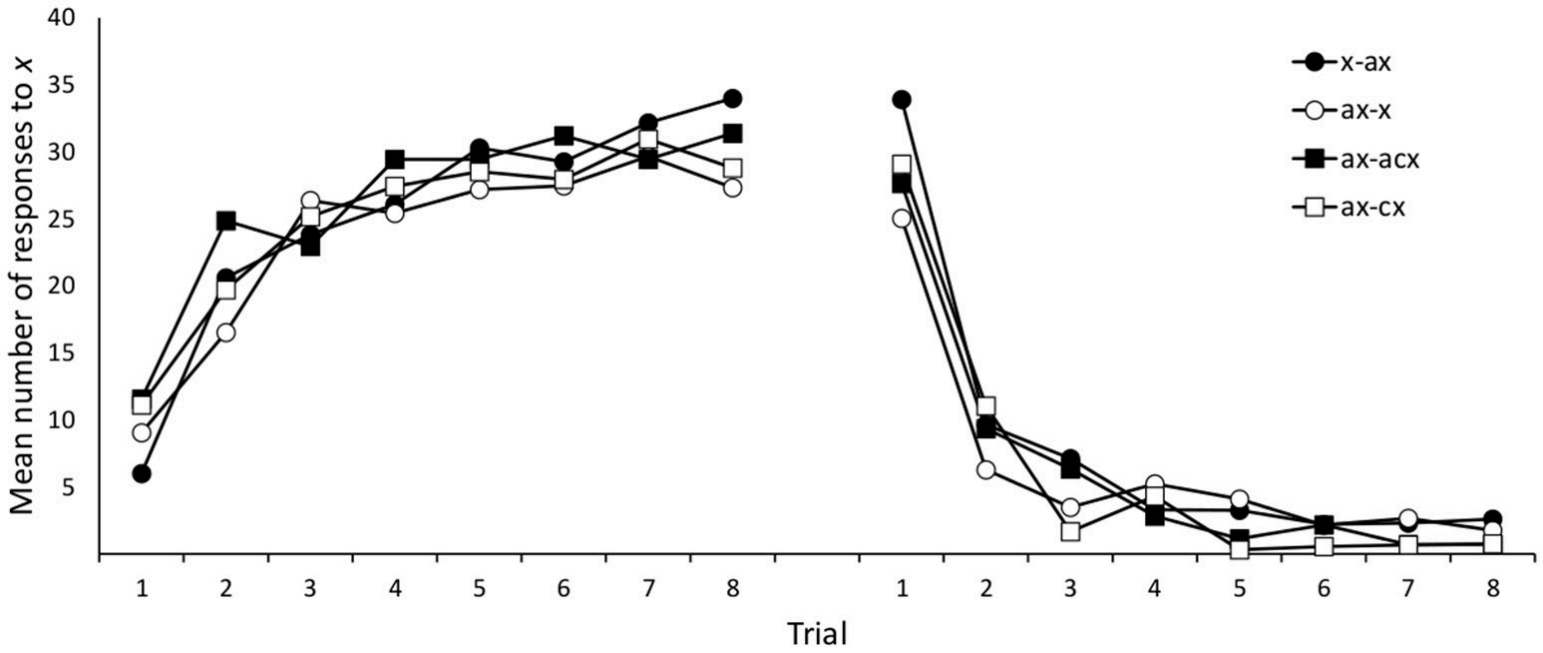
**Mean number of responses to the target cue (***x***) during training in Experiment 2A**.

Nine participants (3, 3, 1, and 2 in groups *x*/*ax, ax*/*x, ax*/*abx, ax*/*bx*, respectively) failed to meet our data selection criteria and were removed from subsequent analyses. Mean responses to cue *x* during training are shown in Figure 4. A 4 (group: *x*/*ax, ax*/*x, ax*/*abx, ax*/*bx*) × 8 (trials) mixed ANOVA on responses to *x* during Phase 1 yielded a significant effect of trial, *F*_(4.62, 406.79)_ = 62.17, *p* < 0.001, η_*p*_^2^ = 0.414, but no significant effect of group, *F* < 1, or group × trial interaction, *F*_(21, 616)_ = 1.240, *p* = 0.211, η_*p*_^2^ = 0.041. Similarly, a 4 (group: *x*/*ax, ax*/*x, ax*/*abx, ax*/*bx*) × 8 (trials) mixed ANOVA on responses to *x* during Phase 2 yielded a significant effect of trial, *F*_(3.70, 325.29)_ = 145.443, *p* < 0.001, η_*p*_^2^ = 0.623, but no effect of group, *F* < 1. The group × trial interaction approached statistical significance, *F*_(21, 616)_ = 1.442, *p* = 0.092, η_*p*_^2^ = 0.047.

**Figure 5:**
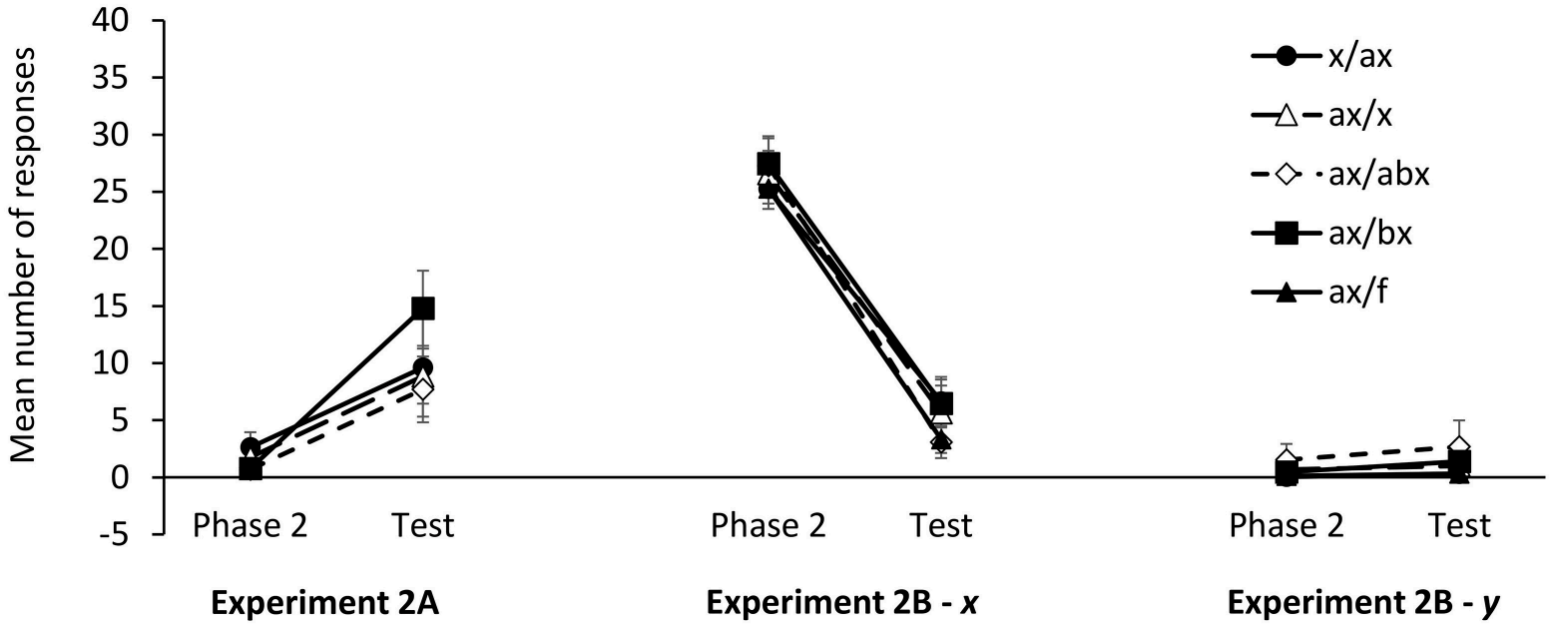
**Mean number of responses at test in Experiments 2A–2B**. Error bars denote standard errors of the mean.

As explained above, in the present experiment we did not use a control group to assess the recovery of information from Phase 1. Instead, we measured response recovery within-participants by comparing responses to *x* during the last trial of Phase 2 and at test. The mean number of responses during the last Phase 2 trial and at test is depicted in Figure 5. Although, the pattern of results suggests that the recovery of first-learned information was stronger in group *ax*/*bx*, a 2 (stage: Phase 2 vs. Test) × 4 (group: *x*/*ax, ax*/*x, ax*/*abx, ax*/*bx*) ANOVA failed to detect a significant stage × group interaction, *F*_(3, 88)_ = 1.49, *p* = 0.233, η_*p*_^2^ = 0.047. Only the main effect of stage reached statistical significance, *F*_(1, 88)_ = 34.44, *p* < 0.001, η_*p*_^2^ = 0.281. Paired sample *t*-tests showed that the mean number of responses at tests was larger than the mean number of responses at the end of Phase 2 in all groups, smallest *t*_(22)_ = 2.15, *p* = 0.043, *d*_*z*_ = 0.45.

## Experiment 2B

In all the previous experiments, the retrieval of the information learned in the first stage was assessed by measuring whether participants responded to the target cue in a manner consistent with the *x-o1* association trained in that phase. In Experiment 2B the target cue was paired with *o2* during Phase 1 and with *o1* during Phase 2. In this case, we expected that the recovery of the information learned in the first stage would give rise to a decrease in the expectancy of *o1* and, consequently, to a decrease in responding to cue *x*. This modification allowed us to check that the results observed in the previous experiments cannot be attributed to the specific outcomes used in Phases 1 and 2.

Additionally, in Experiment 2B we explored whether changing the configuration of the target cue at test also induced a recovery in responding to another cue, *y*, which had also been paired with a different outcome in each phase. If the change in the configuration of *x* creates something analogous to a context change, then one might expect this manipulation to have an effect on responding to any other cue presented in the new context. Alternatively, the effects of the manipulation might be restricted to the cue that experienced the configuration change, having not impact whatsoever on other cues.

### Method

#### Participants and apparatus

One hundred and forty five psychology students from the University of Deusto participated in exchange for course credit. All of them were tested in the same cubicles used in Experiments 1B and 1C. Random allocation of participants to experimental conditions resulted in 34 participants in group *x*/*ax*, 24 in group *ax*/*x*, 29 in group *ax*/*bx*, 26 in group *ax*/*abx*, and 32 in group *ax*/*f*.

#### Procedure and design

The procedure was identical to previous experiments. The design of the experiment, shown in Table 5, was largely inspired in the design of Experiment 2A. The main difference is that cue (*a*)*x* was paired with outcome *o2* in Phase 1 and with outcome *o1* in Phase 2. Therefore, in this experiment, participants' responses reflect an expectation of the outcome trained in Phase 2. Another crucial difference between both experiments is that in addition to *x*, participants were also trained in another cue, *y*, which was also paired with a different outcome in each phase. This allowed us to test whether changing the configuration of cue *x* at test not only affected responding to *x* itself but also to other cues that had been used in an interference treatment. Finally, to measure the baseline level of responding to *y* at test, we included a fifth group, *ax*/*f*, that was not tested with *x*.

**Table 5:** **Design summary of Experiment 2B**.

	**Training**			
**Group**	**Phase 1**	**Phase 2**	**Test 1**	**Test 2**
x/ax	**x-o2 (8)**	**x-o1 (8)**	**ax?**	**y?**
	**y-o1 (8)**	**y-o2 (8)**		
	c-o1 (4)	c-o1 (4)		
	de-o2 (4)	de-o2 (4)		
ax/x	**ax-o2 (8)**	**ax-o1 (8)**	**x?**	**y?**
	**y-o1 (8)**	**y-o2 (8)**		
	c-o1 (4)	c-o1 (4)		
	de-o2 (4)	de-o2 (4)		
ax/bx	**ax-o2 (8)**	**ax-o1 (8)**	**bx?**	**y?**
	**y-o1 (8)**	**y-o2 (8)**		
	c-o1 (4)	c-o1 (4)		
	de-o2 (4)	de-o2 (4)		
ax/abx	**ax-o2 (8)**	**ax-o1 (8)**	**abx?**	**y?**
	**y-o1 (8)**	**y-o2 (8)**		
	c-o1 (4)	c-o1 (4)		
	de-o2 (4)	de-o2 (4)		
ax/f	**ax-o2 (8)**	**ax-o1 (8)**	**f?**	**y?**
	**y-o1 (8)**	**y-o2 (8)**		
	c-o1 (4)	c-o1 (4)		
	de-o2 (4)	de-o2 (4)		

*Cues a-f and x denote different colors in the Spy-Radio, counterbalanced across participants. Outcomes o1 and o2 denote the absence or presence of mines in the road, respectively. The numbers in parentheses denote the number of times each type of trial was presented within a specific phase. Bold characters denote trials involving the target cues*.

### Results and discussion

**Figure 6:**
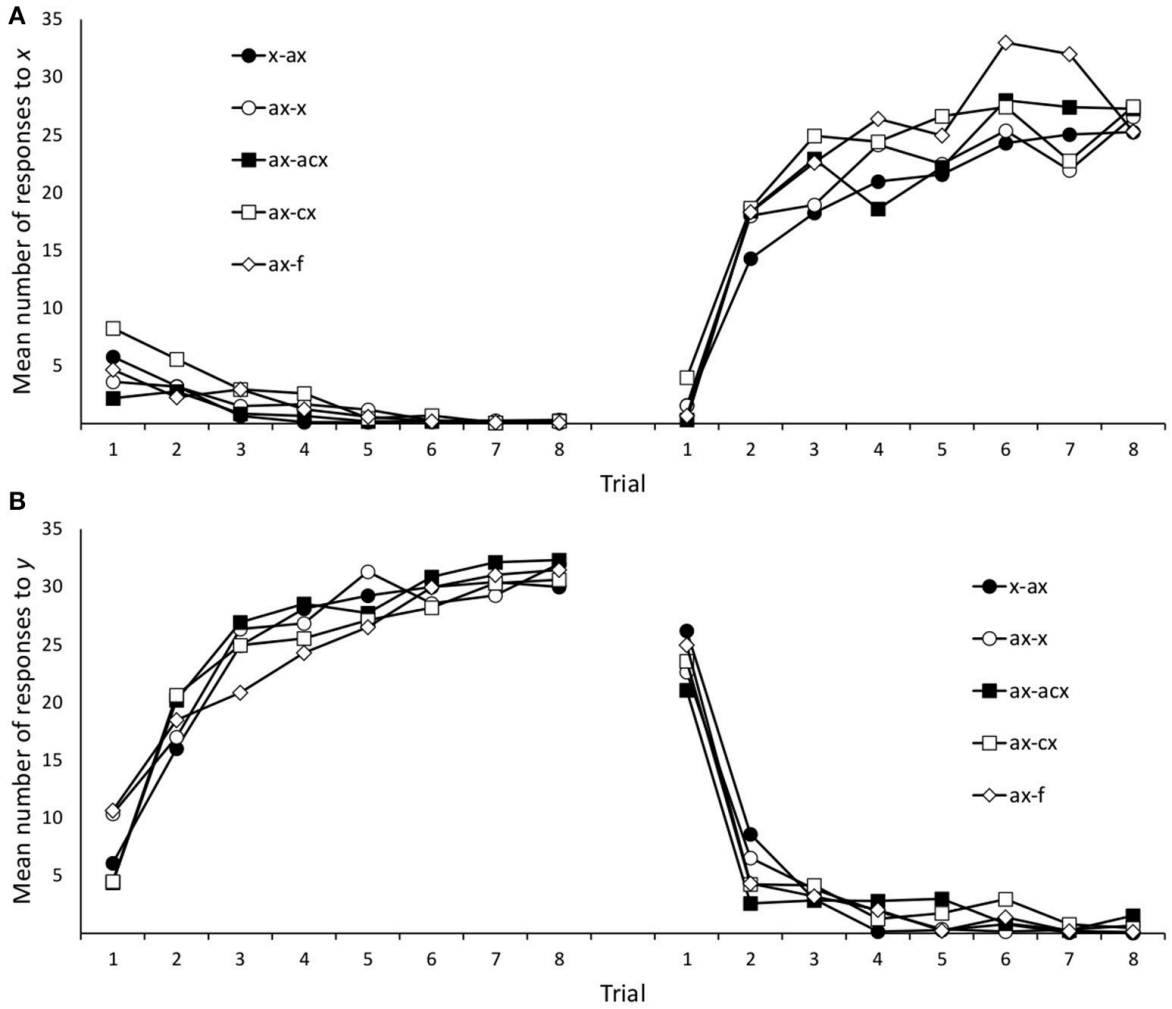
**Mean number of responses to the target cues (***x***/***y***) during training in Experiment 2B**. Panels **(A,B)** refer to cue *x* and *y*, respectively.

Data from 38 participants (10, 5, 5, 11, and 7, from groups *x*/*ax, ax*/*x, ax*/*bx, ax*/*abx*, and *ax*/*f*, respectively) were removed from the analyses following the same selection criteria used in the previous experiments. Figure 6 depicts mean responses for the two target cues, *x* and *y*, during both training phases. As in previous experiments, we explored these responses by means of separate group × trial ANOVAs for each Phase and cue. Only the main effect of trials reached statistical significance in these ANOVAs, smallest *F*_(2.70, 275.60)_ = 13.37, *p* < 0.001, η_*p*_^2^ = 0.116. The other main effects and interactions were non-significant in all the ANOVAs, largest *F*_(28, 714)_ = 1.25, *p* = 0.173, η_*p*_^2^ = 0.047.

As in Experiment 2A, the analysis of responses to cues *x* and *y* at test focused on the difference between the number of responses to the target cues in the last Phase 2 trial and the number of responses in the test trial. The mean number of responses in the last trial of Phase 2 and at test are depicted in Figure 5. A 2 (stage: Phase 2 vs. Test) × 4 (group: *x*/*ax, ax*/*x, ax*/*abx, ax*/*bx*) ANOVA on the number of responses to *x* failed to detect a significant stage × group interaction, *F* < 1. Only the main effect of stage was statistically significant, *F*_(1, 78)_ = 206.3, *p* < 0.001, η_*p*_^2^ = 0.726. A series of paired-samples *t*-tests confirmed that the mean number of responses at test was significantly different from the mean number of responses in the last Phase 2 trial in all groups, smallest *t*_(18)_ = 5.92, *p* < 0.001, *d*_*z*_ = 1.36.

A similar 2 (stage: Phase 2 vs. Test) × 5 (group: *x*/*ax, ax*/*x, ax*/*abx, ax*/*bx, ax/f*) ANOVA on responses to *y* also failed to find a significant stage × group interaction, *F*_(4, 102)_ < 1, although it did detect a main effect of stage, *F*_(1, 102)_ = 4.42, *p* = 0.038, η_*p*_^2^ = 0.042. A series of paired-samples *t*-tests revealed that the number of responses at the end of Phase 2 and Test were significantly different only in group *x*/a*x, t*_(23)_ = 2.77, *p* = 0.011, *d*_*z*_ = 0.64, and marginally significant in group *ax*/*f*, *t*_(24)_ = 2.00, *p* = 0.057, *d*_*z*_ = 0.41. Given that the ANOVA failed to find any significant stage × group interaction, the fact that individual *t*-tests yielded significant results in some cases but not in other must be attributed to random variance. Furthermore, the largest effect sizes among responses to cue *y* (*d*_*z*_ = 0.64) are noticeably smaller than the smallest effect sizes among responses to cue *x* (*d*_*z*_ = 1.36).

These results show that, as in previous experiments, a change in the configuration of the trained cue(s) at test produced a decrease in responding consistent with the information learned in the second stage of an interference treatment. However, this change in responding affected mainly the cue whose configuration had change (i.e., *x*) and it did not transfer so reliably to a second cue that had also undergone an interference treatment (i.e., *y*).

## General discussion

In Experiments 1A–1C participants were trained in an interference paradigm and some of them were tested with only one half of the compound cue that had been presented during training. The results of the three experiments confirmed that this change in the cue configuration reduced the amount of interference relative to a control condition in which participants were trained and tested with the same cue configuration. Experiments 2A and 2B demonstrated that a similar attenuation of retroactive interference is observed with different types of configural manipulations: The same effects were observed either removing, adding, or replacing elements from the cue configuration. Together with previous experiments (Vervliet et al., 2004, 2005), these results support the idea that the response recovery effects typically induced by contextual manipulations can also be observed in response to changes in the configuration of the target cues.

An important difference between the present series of experiments and the set of experiments conducted by Vervliet et al. (2004, 2005) is that the manipulation conducted in the present study was analogous to an AAB renewal design, while the design used by Vervliet et al. was analogous to ABA renewal. That is, in our experiments the configuration of the cue was the same in both training phases, but different at test. In contrast, in the experiments conducted by Vervliet et al. the configuration of the cue was the same during Phase 1 and at test, but different during Phase 2. This difference is not trivial, because AAB renewal is weaker than AAB (Nakajima et al., 2000; Tamai and Nakajima, 2000; Thomas et al., 2003) and also more difficult to explain within the most traditional associative framework.

The most popular associative models (e.g., Rescorla and Wagner, 1972) assume that learning new contingencies involves the unlearning of previous associations. For instance, in an interference design, participants would need to erase the *x-o1* association to learn the *x-o2* association. In general, information retrieval effects like renewal are highly problematic for these models, as they show that the original *x-o1* association remains functional and can be expressed in the appropriate conditions. However, ABA renewal, in particular, does not pose a daunting challenge for these models. As the context, or the configuration of the cue, is different in Phase 1 and Phase 2, it can always be assumed that the new features of the context or the cue (B) in Phase 2 take up any change in associative strength, leaving the original memory trace (A) intact. AAB renewal, in contrast, poses a fundamental problem for traditional models of learning.

By far, the most successful and popular explanation for renewal and other response-recovery effects is the model proposed by Bouton (1993, 1994). According to this model, the cue-outcome association learned during the first stage of an interference experiment (i.e., *x-o1* in our experiments) is always context-independent. When participants are later faced with contradictory information during the second stage (i.e., *x-o2*) an inhibitory association between the cue and the older outcome (*o1*) must be learned to overcome the influence of previous learning. In Bouton's model, this new inhibitory association is assumed to be context-dependent, which means that it is only functional if the cue is presented under the same circumstances that took place originally while the conflicting information was being learned. Therefore, if anything changes between the stage in which the conflicting information was learned (i.e., Phase 2 in our experiments) and test, then the responses appropriate with the original learning (i.e., Phase 1) will reappear. This assumption allows Bouton's model to explain successfully all types of renewal (ABA, AAB, and ABC).

Although, Bouton's model is focused on the role of contexts in the retrieval of conflicting information, it has long been known that changes in physical context are not the only factors that facilitate the retrieval of first-learned information. For instance, classical conditioning studies show that the mere passage of time can induce the recovery of a previously extinguished conditioned response (Pavlov, 1927; Rosas et al., 2001). Similarly, the mere presentation of the unconditioned stimulus immediately after extinction is known to reinstate conditioned responses (Rescorla and Heth, 1975; Vila and Rosas, 2001). Bouton's model can accommodate these findings by assuming that they do also involve a change in the (subjective) context. More recently, human contingency learning experiments have shown that procedural details like the instructions provided to participants, the frequency and type of responses requested, and the presence of filler trials can also modulate the retrieval of first- or second-learned information in interference paradigms (Matute et al., 2002, 2011; Vadillo et al., 2004; Rosas et al., 2006a). The results of our experiments and those conducted by Vervliet et al. (2004, 2005) suggests that the configuration of the target cues might also have the functional properties of a context change (see also, Bouton and Swartzentruber, 1991; Rowe and Craske, 1998; Lovibond et al., 2000).

Our results also pose interesting problems for configural learning theories (Pearce, 1987, 1994). These models describe how learning about one cue generalizes to similar configurations of cues. The configural model developed by Pearce (1994) is not well suited to explain interference phenomena, because it shares with traditional models (e.g., Rescorla and Wagner, 1972) the assumption that adaptation to new contingencies is achieved by means of catastrophic forgetting or “unlearning” of the old ones. In principle, the earlier version of this model proposed by Pearce (1987) is better equipped to deal with the present data, because it allows for the independent storage of excitatory and inhibitory links. In other words, conflicting associations, like the ones learned in Phases 1 and 2 of the present studies, can coexist in the cognitive system. However, to provide a complete explanation of our results, it would also be necessary to implement the assumption that first-learned associations (*ax-o1* in Experiments 1A–1C) generalize more efficiently to similar configurations than second-learned associations (*ax-o2* in Experiments 1A–1C; see Nelson, 2002).

From a different theoretical perspective, an interesting feature of our Experiments 1A–1C is that they involve the counteraction of two effects that, when presented alone, are known to reduce responding, namely, extinction and overshadowing. That is to say, participants responded more to a cue that was both overshadowed and extinguished than to a cue that was either overshadowed or extinguished. Although counterintuitive, similar counteractive effects have been reported between, for example, overshadowing and latent inhibition, overshadowing and degraded contingency, or cue-interference and degraded contingency (Blaisdell et al., 1998; Urcelay and Miller, 2006; Wheeler and Miller, 2007). These effects pose insurmountable problems for the vast majority of learning models. However, they are successfully accounted for by the extended comparator hypothesis (Miller and Matzel, 1988; Denniston et al., 2001; Stout and Miller, 2007). Without entering into the details of the model, the theory assumes that a treatment (e.g., latent inhibition) that reduces responding to a cue also reduces the potential of that cue to interfere with responding to a second cue. In the case of our experiments, extinguishing cue *a* not only reduces responding to cue *a* itself, but also reduces cue *a*'s ability to overshadow *x*. Therefore, paradoxically, responses to cue *x* might reappear because it is both overshadowed and extinguished. Although intriguing, this model is unable to account for the results of Experiments 2A and 2B, where our manipulation of cue configuration is less amenable to an interpretation in terms of cue competition effects, like overshadowing.

In a recent series of studies we have found that unexpected changes in cue-outcome contingency can induce a change in the amount of attention allocated to cues and contexts. For instance, participants tested by Vadillo et al. (2016) paid more attention to the experimental context after a sudden reversal of cue-outcome contingencies. According to Luque et al. (2016), these shifts in attention result from a rapid exploratory process directed at processing any feature of the experimental situation that might disambiguate the meaning of cues and thus avoid future prediction errors (see also Kruschke, 2001; Rosas et al., 2006a). Based on those studies, we can further speculate that a similar shift in attention may have taken place in the present experiments. If participants' attention shifts more often from one cue to another after experiencing a prediction error (i.e., at the beginning of Phase 2 in the present studies), the additional exploration of both cues could result in a qualitatively different representation of the compound. Perhaps, one in which the cues would be better integrated in a distinct configural representation. If this were the case, this would make responding more susceptible to be disrupted by changes in the configuration of the cues.

An important shortcoming of the present series of experiments is that the intermediate level of responding at test cannot be unambiguously attributed to the recovery of the first-learned association. As can be seen in Figures 2, 4, 5, participants also showed some moderate level of responding in the first trials of Phase 1, that is, before they could possibly know which outcome follows each cue. This suggests that a moderate number of responses could be due either to some reactivation of the associations learned during Phase 1 or, alternatively, to simple uncertainty about the outcome that will follow a cue. It is important to note that this shortcoming does not affect just the present study, but is a general limitation of many experiments that have explored information retrieval in human predictive learning tasks (e.g., Pineño and Miller, 2004; Rosas and Callejas-Aguilera, 2006) and also in animal experiments (e.g., Thomas et al., 2003). In any case, we think that there are objective reasons to believe that our results involve, at least in part the recovery of responding consistent with Phase 1 learning. If participants' responses at test were only driven by uncertainty, we would expect a similar level of responding regardless of the specific order in which *x-o1* and *x-o2* associations were trained. However, the results of Experiments 2A and 2B suggest that this is not the case. Figure 5 shows that responding to *x* was higher in Experiments 2A, than in Experiment 2B. A *post-hoc t*-test (corrected for unequal variances) comparing responding to *x* in these two experiments yielded a marginally significant difference, *t*_(177.69)_ = 1.811, *p* = 0.072, *d*_*s*_ = 0.25, 95% CI [−0.02, 0.52]. Although this evidence is admittedly weak and inconclusive, it does suggest that a genuine primacy effect might be present in our data.

Most associative models of learning consider that punctuate cues differ from physical contexts in several ways. For example, within the Rescorla-Wagner model, the context can be represented as a set of constant and low-salience stimuli that are weakly associated with the outcome. In contrast, cues are highly salient and have the potential to develop strong links with the outcome (see also Mackintosh, 1975; Archer and Sjödén, 1979). Other models differentiate between cues and contexts attending to their associative status: cues are directly associated with the outcome, while contexts modulate the cue-outcome association (Bouton, 1994). A third class of models (e.g., Logan, 1988; Howard and Kahana, 2002) does not differentiate between cues and contexts at all and assumes that all the stimuli presented in the experimental situation are entangled in a single representation. Setting aside the specific predictions of these models, this general idea provides an interesting framework to understand how changes in the experimental situation can modulate responding, regardless of whether they refer to changes in cues, contexts or other factors. Additional support for this latest proposal has been provided by Matute et al. (2011) using an experimental paradigm similar to the one used herein.

Beyond their theoretical interpretation, it seems clear that the results of the present series of experiments suggest that changing the cue configuration can attenuate the effects of interference. In this way, they provide further support to the idea that interference does not result in the unlearning of previous knowledge and that interfered information can still influence behavior under appropriate conditions. Our results also extend the conclusions of earlier studies by showing that a change in the configuration of the cue (e.g., removing, adding or replacing elements from a compound cue) is one of the manipulations that can give rise to the expression of interfered information.

## Author contributions

All authors developed the study concept. CC developed the software, conducted the experiments, and analyzed the data. CC drafted the manuscript and MV and HM provided critical revisions. All authors approved the final version of the manuscript for submission. The experiments reported in this article were conducted as part of CC's doctoral dissertation under the supervision of HM and MV while the three authors worked at the University of Deusto.

## Funding

This research was supported by Dirección General de Investigación of the Spanish Government (Grant No. PSI2016-78818-R) to HM.

### Conflict of interest statement

The authors declare that the research was conducted in the absence of any commercial or financial relationships that could be construed as a potential conflict of interest.

## References

[B1] American Psychological Association (2002). Ethical Principles of Psychologists and Code of Conduct. Washington, DC: Author.

[B2] Anderson M. C. Bjork R. A. Bjork E. L. (1994). Remembering can cause forgetting: retrieval dynamics in long-term memory. J. Exp. Psychol. Learn. 20, 1063–1087. 10.1037/0278-7393.20.5.1063 7931095

[B3] Archer T. Sjödén P. O. (1979). Neophobia in taste aversion conditioning: individual differences and effects of contextual changes. Physiol. Psychol. 7, 364–369. 10.3758/BF03326657

[B4] Baum M. Jacobs W. J. (1989). Loud noise potentiates conditioned fear in extinction using a CER (lick suppression) paradigm in rats. Bull. Psychon. Soc. 27, 449–451. 10.3758/BF03334652

[B5] Blaisdell A. P. Bristol A. S. Gunther L. M. Miller R. R. (1998). Overshadowing and latent inhibition counteract each other: support for the comparator hypothesis. J. Exp. Psychol. Anim. B. 24, 335–351. 10.1037/0097-7403.24.3.335 9679309

[B6] Bouton M. E. (1993). Context, time, and memory retrieval in the interference paradigms of Pavlovian conditioning. Psychol. Bull. 114, 80–99. 10.1037/0033-2909.114.1.80 8346330

[B7] Bouton M. E. (1994). Conditioning, remembering, and forgetting. J. Exp. Psychol. Anim. B. 20, 219–231. 10.1037/0097-7403.20.3.219

[B8] Bouton M. E. Bolles R. C. (1979). Contextual control of the extinction of conditioned fear. Learn. Motiv. 10, 445–466. 10.1016/0023-9690(79)90057-2

[B9] Bouton M. E. Peck C. A. (1992). Spontaneous recovery in cross-motivational transfer (counter-conditioning). Anim. Learn. Behav. 20, 313–321. 10.3758/BF03197954

[B10] Bouton M. E. Ricker S. T. (1994). Renewal of extinguished responding in a second context. Anim. Learn. Behav. 22, 313–324. 10.3758/BF03209840

[B11] Bouton M. E. Swartzentruber D. (1991). Sources of relapse after extinction in Pavlovian and instrumental learning. Clin. Psychol. Rev. 11, 123–140. 10.1016/0272-7358(91)90091-8

[B12] De la Casa G. Lubow R. E. (1995). Latent inhibition in conditioned taste aversion: the roles of stimulus frequency and duration and the amount of fluid ingested during preexposure. Neurobiol. Learn. Mem. 64, 125–132. 10.1006/nlme.1995.1051 7582820

[B13] Denniston J. C. Savastano H. I. Miller R. R. (2001). The extended comparator hypothesis: learning by contiguity, responding by relative strength, in Handbook of Contemporary Learning Theories, eds Mower R. R. Klein S. B. (Mahwah, NJ: Erlbaum), 65–117.

[B14] Escobar M. Pineño O. Matute H. (2002). A comparison between elemental and compound training of cues in retrospective revaluation. Anim. Learn. Behav. 30, 228–238. 10.3758/BF03192832 12391789

[B15] Howard M. W. Kahana M. J. (2002). A distributed representation of temporal context. J. Math. Psychol. 46, 269–299. 10.1006/jmps.2001.1388

[B16] Kamin L. J. (1968). Attention-like processes in classical conditioning, in Miami Symposium on the Prediction of Behavior, 1967: Aversive Stimulation, ed Jones M. R. (Coral Gables, FL: University of Miami Press), 9–31.

[B17] Kruschke J. K. (2001). Toward a unified model of attention in associative learning. J. Math. Psychol. 45, 812–863. 10.1006/jmps.2000.1354

[B18] Lewandowsky S. (1991). Gradual unlearning and catastrophic interference: a comparison of distributed architectures, in Relating Theory and Data: Essays on Human Memory in Honor of Bennet B. Murdock, eds Hockley W. E. Lewandowsky S. (Hillsdale, NJ: Lawrence Erlbaum Associates, Inc), 445–476.

[B19] Logan G. D. (1988). Toward an instance theory of automatization. Psychol. Rev. 95, 492–527. 10.1037/0033-295X.95.4.492

[B20] Lovibond P. F. Davis N. R. O'Flaherty A. S. (2000). Protection from extinction in human fear conditioning. Behav. Res. Ther. 38, 967–983. 10.1016/S0005-7967(99)00121-7 11004736

[B21] Lubow R. E. (1973). Latent inhibition. Psychol. Bull. 79, 398–407. 10.1037/h0034425 4575029

[B22] Luque D. Morís J. Cobos P. L. López F. J. (2009). Interference between cues of the same outcome in a non-causally framed scenario. Behav. Process. 81, 328–332. 10.1016/j.beproc.2008.11.009 19070656

[B23] Luque D. Morís J. Orgaz C. Cobos P. L. Matute H. (2011). Backward blocking and interference between cues are empirically equivalent in non-causally framed learning tasks. Psychol. Rec. 61, 141–152.

[B24] Luque D. Vadillo M. A. Le Pelley M. E. Beesley T. (2016). Prediction and uncertainty in associative learning: examining controlled and automatic components of learned attentional biases. Q. J. Exp. Psychol.. [Epub ahead of print]. 10.1080/17470218.2016.1188407 27174735

[B25] Mackintosh N. J. (1975). A theory of attention: variations in the associability of stimuli with reinforcement. Psychol. Rev. 82, 276–298. 10.1037/h0076778

[B26] Matute H. Lipp O. V. Vadillo M. A. Humphreys M. S. (2011). Temporal contexts: filling the gap between episodic memory and associative learning. J. Exp. Psychol. Gen. 140, 660–673. 10.1037/a0023862 21744983

[B27] Matute H. Vadillo M. A. Bárcena R. (2007). Web-based experiment control software for research and teaching on human learning. Behav. Res. Methods. 39, 689–693. 10.3758/BF03193041 17958183

[B28] Matute H. Vegas S. De Marez P.-J. (2002). Flexible use of recent information in causal and predictive judgments. J. Exp. Psychol. Learn. 28, 714–725. 10.1037/0278-7393.28.4.714 12109763

[B29] McCloskey M. Cohen N. J. (1989). Catastrophic interference in connectionist networks: the sequential learning problem, in The Psychology of Learning and Motivation, Vol. 24, ed Bower G. H. (San Diego, CA: Academic Press), 109–165.

[B30] Miguez G. Soares J. S. Miller R. R. (2015). The role of test context in latent inhibition of conditioned inhibition: part of a search for general principles of associative interference. Learn. Behav. 43, 228–242. 10.3758/s13420-015-0175-0 25875792 PMC4515373

[B31] Miller R. R. Laborda M. A. Polack C. W. Miguez G. (2015). Comparing the context specificity of extinction and latent inhibition. Learn. Behav. 43, 384–395. 10.3758/s13420-015-0186-x 26100525 PMC4641778

[B32] Miller R. R. Matzel L. D. (1988). The comparator hypothesis: a response rule for the expression of associations, in The Psychology of Learning and Motivation, Vol. 22, ed Bower G. H. (San Diego, CA: Academic Press), 51–92.

[B33] Nakajima S. Tanaka S. Urushihara K. Imada H. (2000). Renewal of extinguished lever-press responses upon return to the training context. Learn. Motiv. 31, 416–431. 10.1006/lmot.2000.1064

[B34] Nelson J. B. (2002). Context specificity of excitation and inhibition in ambiguous stimuli. Learn. Motiv. 33, 284–310. 10.1006/lmot.2001.1112

[B35] Paredes-Olay M. C. Rosas J. M. (1999). Within-subjects extinction and renewal in predictive judgments. Psicológica 20, 195–210.

[B36] Pavlov I. P. (1927). Conditioned Reflexes. London: Oxford University Press.

[B37] Pearce J. M. (1987). A model for stimulus generalization in Pavlovian conditioning. Psychol. Rev. 94, 61–73. 10.1037/0033-295X.94.1.61 3823305

[B38] Pearce J. M. (1994). Similarity and discrimination: a selective review and a connectionist model. Psychol. Rev. 101, 587–607. 10.1037/0033-295X.101.4.587 7984708

[B39] Pineño O. Miller R. R. (2004). Signaling a change in cue-outcome relations in human predictive judgments. Learn. Behav. 32, 360–375. 10.3758/BF03196034 15672830

[B40] Pineño O. Ortega N. Matute H. (2000). The relative activation of the associations modulates interference between elementally-trained cues. Learn. Motiv. 31, 128–152. 10.1006/lmot.1999.1047

[B41] Raes A. K. De Raedt R. (2012). The effect of counterconditioning on evaluative responses and harm expectancy in a fear conditioning paradigm. Behav. Ther. 43, 757–767. 10.1016/j.beth.2012.03.012 23046778

[B42] Ratcliff R. (1990). Connectionist models of recognition memory: constraints imposed by learning and forgetting functions. Psychol. Rev. 97, 285–308. 10.1037/0033-295X.97.2.285 2186426

[B43] Rescorla R. A. Heth C. D. (1975). Reinstatement of fear to an extinguished conditioned stimulus. J. Exp. Psychol. Anim. B. 1, 88–96. 10.1037/0097-7403.1.1.88 1151290

[B44] Rescorla R. A. Wagner A. R. (1972). A theory of Pavlovian conditioning: variations in the effectiveness of reinforcement and nonreinforcement, in Classical Conditioning II: Current Research and Theory, eds Black A. H. Prokasy W. F. (New York, NY: Appelton-Century-Crofts), 64–99.

[B45] Rodríguez G. Márquez R. Gil M. Alonso G. Hall G. (2014). The Hall-Rodríguez theory of latent-inhibition: further assessment of compound stimulus preexposure effects. J. Exp. Psychol. Anim. B. 40, 425–430. 10.1037/xan0000035 25546100

[B46] Rosas J. M. Bouton M. E. (1998). Context change and retention interval can have additive, rather than interactive, effects after taste aversion extinction. Psychon. B. Rev. 5, 79–83. 10.3758/BF03209459

[B47] Rosas J. M. Callejas-Aguilera J. E. (2006). Context switch effects on acquisition and extinction in human predictive learning. J. Exp. Psychol. Learn. Mem. Cogn. 32, 461–474. 10.1037/0278-7393.32.3.461 16719659

[B48] Rosas J. M. Callejas-Aguilera J. E. Ramos-Álvarez M. M. Fernández Abad M. J. (2006a). Revision of retrieval theory of forgetting: what does make information context-specific. Intern. Jour. Psych. Psychol. Therapy. 6, 147–166.

[B49] Rosas J. M. García-Gutiérrez A. Callejas-Aguilera J. E. (2006b). Effects of context change upon retrieval of first and second-learned information in human predictive learning. Psicológica 27, 35–56.

[B50] Rosas J. M. Javier Vila N. Lugo M. López L. (2001). Combined effect of context change and retention interval on interference in causality judgments. J. Exp. Psychol. Anim. B. 27, 153–164. 10.1037/0097-7403.27.2.153 11296490

[B51] Rowe M. K. Craske M. G. (1998). Effects of varied-stimulus exposure training on fear reduction and return of fear. Behav. Res. Ther. 36, 719–734. 10.1016/S0005-7967(97)10017-1 9682527

[B52] Soltysik S. S. Wolfe G. E. Nicholas T. Wilson W. J. Garcia-Sanchez J. L. (1983). Blocking of inhibitory conditioning within a serial conditioned stimulus-conditioned inhibitor compound: maintenance of acquired behavior without an unconditioned stimulus. Learn. Motiv. 14, 1–29. 10.1016/0023-9690(83)90010-3

[B53] Stout S. C. Miller R. R. (2007). Sometimes-competing retrieval (SOCR): a formalization of the comparator hypothesis. Psychol. Rev. 114, 759–783. 10.1037/0033-295X.114.3.759 17638505

[B54] Tamai N. Nakajima S. (2000). Renewal of formerly conditioned fear in rats after extensive extinction training. Int. J. Comp. Psychol. 13, 137–147.

[B55] Thomas B. L. Larsen N. Ayres J. J. B. (2003). Role of context similarity in ABA, ABC, and AAB renewal paradigms: implications for theories of renewal and for treating human phobias. Learn. Motiv. 34, 410–436. 10.1016/S0023-9690(03)00037-7

[B56] Urcelay G. P. Miller R. R. (2006). Counteraction between over-shadowing and degraded contingency treatments: support for the extended comparator hypothesis. J. Exp. Psychol. Anim. B. 32, 21–32. 10.1037/0097-7403.32.1.21 PMC1796739 16435962

[B57] U.S. Department of Health Human Services (2009). Code of Federal Regulations, Title 45, Public Welfare, Part 46: Protection of Human Subjects. Washington, DC: Author.

[B58] Vadillo M. A. Bárcena R. Matute H. (2006). The internet as a research tool in the study of associative learning: an example from overshadowing. Behav. Process. 73, 36–40. 10.1016/j.beproc.2006.01.014 16522356

[B59] Vadillo M. A. Castro L. Matute H. Wasserman E. A. (2008). Backward blocking: the role of within-compound associations and interference between cues trained apart. Q. J. Exp. Psychol. 61, 185–193. 10.1080/17470210701557464 17886193

[B60] Vadillo M. A. Orgaz C. Luque D. Cobos P. L. López F. J. Matute H. (2013). The role of outcome inhibition in interference between outcomes: a contingency-learning analogue of retrieval-induced forgetting. Brit. J. Psychol. 104, 167–180. 10.1111/j.2044-8295.2012.02110.x 23560664

[B61] Vadillo M. A. Orgaz C. Luque D. Nelson J. B. (2016). Ambiguity produces attention shifts in category learning. Learn. Mem. 23, 134–140. 10.1101/lm.041145.115 26980780 PMC4793202

[B62] Vadillo M. A. Vegas S. Matute H. (2004). Frequency of judgment as a context-like determinant of predictive judgments. Mem. Cogn. 32, 1065–1075. 10.3758/BF03196882 15813490

[B63] Vervliet B. Vansteenwegen D. Baeyens F. Hermans D. Eelen P. (2005). Return of fear in a human differential conditioning paradigm caused by a stimulus change after extinction. Behav. Res. Ther. 43, 357–371. 10.1016/j.brat.2004.02.005 15680931

[B64] Vervliet B. Vansteenwegen D. Eelen P. (2004). Generalization of extinguished skin conductance responding in human fear conditioning. Learn. Memory. 11, 555–558. 10.1101/lm.77404 15466308

[B65] Vervliet B. Vansteenwegen D. Hermans D. Eelen P. (2007). Concurrent excitors limit the extinction of conditioned fear in humans. Behav. Res. Ther. 45, 375–383. 10.1016/j.brat.2006.01.009 16597433

[B66] Vila N. J. Rosas J. M. (2001). Reinstatement of acquisition performance by the presentation of the outcome after extinction in causality judgments. Behav. Process. 56, 147–154. 10.1016/S0376-6357(01)00197-8 11738508

[B67] Wheeler D. S. Miller R. R. (2007). Interactions between retroactive interference and context-mediated treatments that impair Pavlovian conditioned responding. Learn. Behav. 35, 27–35. 10.3758/BF03196071 17464367 PMC1857297

